# Successful Smoking Cessation in COPD: Association with Comorbidities and Mortality

**DOI:** 10.1155/2012/725024

**Published:** 2012-11-14

**Authors:** H. Kupiainen, V. L. Kinnula, A. Lindqvist, D. S. Postma, H. M. Boezen, T. Laitinen, M. Kilpeläinen

**Affiliations:** ^1^Department of Pulmonary Diseases and Clinical Allergology, Turku University Hospital and University of Turku, Kiinamyllynkatu 13, 20520 Turku, Finland; ^2^Clinical Research Unit for Pulmonary Diseases and Division of Pulmonology, Helsinki University Central Hospital, Tukholmankatu 8C, 00290 Helsinki, Finland; ^3^Department of Pulmonology, University Medical Center Groningen, Hanzeplein 1, 9700 RB Groningen, The Netherlands; ^4^Department of Epidemiology, University Medical Center Groningen, Hanzeplein 1, 9700 RB Groningen, The Netherlands

## Abstract

Smoking cessation is the cornerstone of COPD management, but difficult to achieve in clinical practice. The effect of comorbidities on smoking cessation and risk factors for mortality were studied in a cohort of 739 COPD patients recruited in two Finnish University Hospitals. The diagnosis of COPD was done for the first time on average 5.5 years prior to the enrollment. Data from the medical records and followup questionnaires (years 0, 1, 2, and 4) have been analyzed. The patients' lung function varied greatly; mean FEV_1_ 58% of predicted. A total of 60.2% of men and 55.6% of women had been able to quit smoking. Alcohol abuse (OR 2.1, 95% CI 1.4–3.3) and psychiatric conditions (OR 1.8, 95% CI 1.2–2.7) were strongly related to low success rates of quitting. Among current smokers high nicotine dependency was again explained by alcohol abuse and psychiatric conditions. Non-quitters were younger than quitters, but their mortality rates remained significantly higher even when the model was adjusted for impairment of lung functions and comorbidities. In conclusion, co-existing addiction and psychiatric diseases significantly decreased the success rates in smoking cessation and increased mortality among the patients.

## 1. Introduction 

Smoking is by far the strongest risk factor of COPD. Smoking cessation has been shown to decelerate the progression of the disease and reduce mortality [1, 2]. In addition, smoking cessation is associated with a significant reduction of COPD exacerbations [3] and hospital admissions [4]. Data on predictors of smoking cessation relies almost entirely on population studies [5, 6]. COPD patients on the other hand comprise a heterogeneous group of patients who, in addition to heavy smoking, often display a variety of addictive characteristics, such as alcohol abuse [7] and certain psychiatric disorders [8]. Except for the shown efficacy of cessation medication, such as nicotine-replacement therapy, bupropion, and varenicline [9, 10], there are sparse studies on the clinical characteristics of COPD patients who either succeed or fail in quitting smoking. A previous study has shown that a significant number of COPD patients continued smoking despite of their attempts to quit, underlining the importance of smoking cessation programs in clinical practice [11]. Among Polish smokers simple smoking cessation advice was shown to be more efficient among patients who displayed bronchial obstruction than among smokers with normal lung functions, suggesting that the awareness of COPD may improve the patient's motivation for quitting [12]. In these studies cumulative clinical data from medical records was not available.

We have followed a cohort of 739 COPD patients since the year 2006–07 by gathering their medical records into a single database [13]. The source data covers all health care providers, and the participants' medical history extends up to 5–10 years of prior to the enrollment. The patients are followed for 10 years with the questionnaires mailed every other year, currently the patients are on their fourth follow-up year.

Using this longitudinal clinical and questionnaire data, our aim in the present study is to report the clinical predictors that increase the risk of failure in smoking cessation among COPD patients. Our second aim was to find out whether smoking cessation had an effect on mortality in this hospital-based COPD population that represented all severity stages of the disease.

## 2. Study Subjects and Methods

### 2.1. Study Subjects

Hospital Discharge Registries were used to identify all in- and out-patients who had visited the Pulmonary Clinics of the Helsinki University Central Hospital (HUCH) and Turku University Hospital during the years 1995–2006. The databases were screened by the ICD10 code J44.8 and contained all patients between 18 to 75 years of age. The recruitment of all the identified patients was done through a two-phase mailing campaign. Altogether 844 patients (27%) contacted the researchers by phone and agreed on a visit to the clinical research center in Helsinki or Turku.

The research visits took place in years 2006–07 [13]. The participants gave their written informed consent for collecting and merging their comprehensive medical history from all healthcare providers who had treated them during the past 5–10 years. The participants also agreed to continue their followup through medical records and questionnaires for the next 10 subsequent years. They also donated a blood sample for DNA extraction. The health professionals examined the medical records to confirm the previously given smoking-related COPD or chronic bronchitis diagnosis. The diagnostic procedure described previously in detail [13] was based on clinical outcome, smoking history ≥10 pack years, and spirometry verification of airway obstruction. This evaluation led to the exclusion of a total of 105 patients due to fragmented documentation, insufficient diagnostic testing, and misclassifications.

### 2.2. Smoking-Related Variables

Data regarding the number of pack years and the smoking status were evaluated at two cross sectional time points: at the time of the diagnosis and the enrollment to the current study. The primary source of information for each study subject was the spirometry form. If that data was not available, other time-labeled documents were used. If these were not available, self-reported questionnaire data was used to acquire information about the patient's smoking status. Based on that data, smoking cessation was graded as follows: (1) quitting smoking before the diagnosis of COPD (early quitters), (2) quitting smoking after the diagnosis, either before or during the current study (late quitters), and (3) continued smoking throughout the study (nonquitters). Only the combined results are reported, in case no significant differences between the early and late quitters could be discovered.

### 2.3. Clinical Variables

The reference values for FEV_1_ and FVC used in Finnish clinical practice are validated in large Finnish population samples consisting of males and females with a wide age range [14]. “Cardiovascular diseases” consist of patients diagnosed with one of the following diseases: coronary, cerebrovascular, or peripheral artery occlusive disease diagnosed by an internist. The category “diabetes” includes patients with type 1 and 2 disease. Chronic alcoholism, alcohol use disorder, and treatment of alcohol-use-related disorders were all categorized as “alcohol abuse”. A wide range of psychotic disorders and long-lasting clinical depression and anxiety with a need for regular medication were combined as “psychiatric condition”. The category “cancer” included all malignant solid tumors and malignant haematological diseases. The deaths were verified from the National Death Registry.

### 2.4. Health Related Quality of life and Nicotine Dependence

The summary scores of the participant's general (15D) [15, 16] and airway specific (AQ20) health-related quality of life [16] were obtained at the recruitment visit and from the 1st follow-up questionnaire. Both questionnaires have been validated and standardized internationally [15, 16]. The score of 15D varies from 0 (= dead) to 1 (= full health) and in AQ20 from 0 (= no symptoms or worries over the disease) to 20 (= full range of symptoms and worries over the disease). The patients' nicotine dependence was evaluated with the Fageström nicotine dependency test (FNDT) [17]. Data for this analysis was collected as a part of the fourth year follow-up questionnaire. At that time point 155 patients were still regular smokers. We were able to compute the Fageström's score for 140 of them.

The study approach was approved by the Coordinating Ethics Committee of the Helsinki and Uusimaa Hospital District, and the permission to conduct this research was granted by the Helsinki and Turku University Hospitals.

### 2.5. Statistical Analysis

The statistical software package SPSS (version 18.0) was used to compute differences in distributions of demographic and clinical variables between study groups. Differences in continuous variables were tested using the nonparametric Mann-Whitney U-test and in categorical variables the Chi-square or Fisher exact tests. Multivariate logistic regression model for smoking cessation was created with clinically relevant continuous traits and all comorbidities as independent variables. The dependent variable was quitting smoking either before or after COPD diagnosis, and the independent variables included were gender, age (per year), FEV_1_ (categorized into four classes >80% normal, 65–80% mild, 40–65% moderate, and <40% severe), pack years, alcohol abuse, psychiatric condition, cardiovascular disease, diabetes, and cancer. A similar analysis was done for all-cause mortality: age, gender, smoking cessation, lung functions, and comorbidities were analyzed by univariate analysis and used in a multiple logistic regression model. The Fageström nicotine dependency score was analyzed with linear regression using gender, age, FEV_1_, alcohol abuse, psychiatric conditions requiring medication, diabetes, and cardiovascular diseases as independent variables in the model.

The study approach was approved by the Coordinating Ethics Committee of the Helsinki and Uusimaa Hospital District, and the permission to conduct this research was granted by the Helsinki and Turku University Hospitals.

## 3. Results

### 3.1. Clinical Characteristics of the Cohort

The cohort (*N* = 739) represented smoking-related chronic bronchitis and COPD of all severity stages (Table 1). FEV_1_ ranged from 15 to 100%, mean 58% of predicted [13]. The COPD diagnosis was given for the first time when the participants were on average 58 years of age. During the enrollment to the present study, the average age was 64 years. The participants were diagnosed on average 5.5 years prior to the enrollment, which happened during the years 2006–07, and prospectively they have been followed so far for 4 years. One hundred fourteen patients had deceased during the study (annual mortality 3.8%).

### 3.2. Gender Differences in Smoking Behavior

In the cohort 36% of the participants were women (Table 1). There were no significant differences in age or duration of COPD between genders. However, the number of pack years among men was significantly higher than among women (45 versus 33 pack years, *P* ≤ 0.001). When COPD was first diagnosed, women were more frequently current smokers than men (80% versus 68%, *P* < 0.001). At the time of enrollment to the present study, the difference between genders was diminished (40% of men and 44% of women current smokers).

### 3.3. Quitting History

At the time of the enrollment, a total of 58.6%  (*n* = 433) patients had quitted smoking. 28%  (*n* = 207) of the patients quitted before and 30.6%  (*n* = 226) after the COPD diagnosis was set. Minor, but statistically significant, differences were observed between the groups. The quitters were on average older (65 versus 62 years of age, *P* = 0.001) and reported more pack years (43 versus 38, *P* = 0.011) than nonquitters (Table 1). Their disease was more advanced (FEV_1_ 60.3 versus 64.4% predicted, *P* = 0.004 and FEV_1_/FVC ratio 63.0 versus 66.4, *P* = 0.002).

### 3.4. Comorbidities and Quitting

With respect to co-morbidities alcohol abuse and psychiatric conditions were the most significant risk factors associated with failure in smoking cessation. The prevalence of alcohol abuse (26.6% versus 11.5% *P* values ≤ 0.001) and psychiatric conditions (33.6% versus 17.5%, *P* ≤ 0.001) were both higher among nonquitters than quitters. Cardiovascular diseases were more prevalent among early quitters compared to late or nonquitters, 44.4% and 31.3%, respectively, *P* = 0.005.

Multivariate logistic regression for smoking cessation was analyzed with clinical traits and comorbidities as independent variables (Table 2). Of the comorbid conditions, alcohol abuse and psychiatric conditions remained independent risk factors for failure in smoking cessation. The two conditions were partially overlapping. The prevalence of both conditions was 14.0% (42/299) among nonquitters and 3.1% (13/421) among quitters (*P* < 0.001). Gender or degree of airway obstruction did not contribute in smoking cessation significantly. The older a person was, the greater the chance of success in quitting.

### 3.5. Mortality in the Cohort

Altogether 114 (15.3%) patients had died by June 2011. Mortality rates were significantly higher among nonquitters (20.7%) compared to those among quitters (12.2%, *P* = 0.002). Clinical characteristics that had an effect on mortality are shown in Table 3. In the multivariate model, failure in quitting was strongly associated with increased mortality (OR 2.50, 95% CI 1.55–4.03). Of the comorbidities, cancer (OR 3.08, 95% CI 1.70–5.59) and alcohol abuse (OR 2.03, 95% CI 1.14–3.61) had an independent explanatory role in mortality. Similarly, moderate (OR 3.2, 95% CI 1.3–8.4) and severe (OR 5.6, 95% CI 2.5–17.5) impairment of lung function was associated with increased mortality, but cardiovascular diseases, diabetes, and psychiatric conditions lost the effect in the adjusted model.

### 3.6. Nicotine Dependence among the Current Smokers

Nicotine dependence was evaluated using the Fagerström nicotine dependency test. The mean score among the patients was 4.3 (SD 2.4). The FNDT score did not correlate with FEV_1_ of predicted values. Based on linear regression adjusted with age, gender, FEV_1_, and comorbidities (cardiovascular diseases, diabetes, and cancer), alcohol abuse was the strongest explanatory variable for a high score (beta-value 0.260, *P* = 0.003). Also psychiatric conditions had an independent explanatory value (beta-value 0.183, *P* = 0.04).

## 4. Discussion

In the Finnish elderly population at large, smoking does not exceed 20% among men or 10% among women [18]. In this hospital-based COPD cohort, 40% of men and 44% of women continued smoking on average 5.5 years after their COPD had been diagnosed for the first time. Current smoking was significantly more prevalent among the patients who had alcohol abuse and/or psychiatric conditions. Compared to current smokers, the quitters of the cohort were older, reported more pack years, and had more advanced COPD frequently accompanied by cardiovascular disease, suggesting that the quitting takes place rather late in life and among patients with a disabling disease.

Our findings support and expand the previous findings made on quitting smoking in COPD [11, 12, 19]. In a large questionnaire study (*n* = 58 482) made among the US Veterans, former smokers were older and had more cardiac comorbidities, but better mental health than current smokers [19]. Smoking cessation is strongly recommended in patients with cardiovascular disease and diabetes [20]. In the present study cardiovascular disease seemed to be a strong wake-up call to early smoking cessation and partially explained the better quit rates among males compared to those among females. Diabetes showed not as clear a relationship to smoking cessation, although a similar trend was observed.

COPD patients have been reported to display a twofold risk for depression compared to the general population [21, 22]. Even though presently largely ignored, tobacco dependence among individuals with a mental illness, especially schizophrenia, anxiety and depression, is very high [6, 8, 23, 24]. This might be explained by a robust bidirectional connection between nicotine dependence and vulnerability to psychopathology and susceptibility to certain psychiatric disorders [22]. It has also been reported that heavy drinking impairs success of quitting among COPD patients [11]. In the Finnish population over 50 years of age, hazardous drinking has been reported in 8% of men and 2% of women [25]. In the present COPD cohort the prevalence of alcohol abuse was 1.8 times higher among men and 5 times higher among women and was strongly related to nonquitting.

The Fagerström nicotine dependency test was included in the 4th year follow-up questionnaire and thus gave us information only from the most persistent smokers. The FNDT score of the present COPD cohort was in accordance with previous studies [26]. It has been reported, however, that low FEV_1_ and a high number of pack years indicate strong nicotine dependence [23]. Thus it was somewhat surprising that low FEV_1_ in the present study did not significantly associate with the nicotine dependence. The variance found in FEV_1_ among these long-term persistent smokers is, however, significantly smaller than in general population and thus decreases our power to detect potential association. The association between alcohol abuse and FNDT score in our study further proves the fact that addictive behavior cumulates [27].

Mortality was higher among nonquitters despite of their younger age and better lung functions. The increased mortality was confirmed irrespective of comorbidities, such as cancer or cardiovascular diseases. The fact that quitters are older, have lower lung function, more pack years, and CVD suggests reversed causality; a “healthy smoker” effect in which patients quit smoking only when they are older and have disabling illnesses. However, this does not directly lead to higher mortality rates. It seems that reversed causality is present but is confounded by the addictive profile of nonquitters with physical and mental problems and death at earlier age [2].

In the present study all the COPD patients (with the recruitment rate of 27%) treated in the clinic were invited to the study without further selection. Often in corresponding study designs the patient cohort has been rather small [28] or excluded relevant subgroups, such as patients with certain comorbidities, addictions, or obesity [29]. In randomized clinical trials, combined pharmacotherapy and counseling seem to lead to best long-term abstinence rates [9]. Participants' compliance, however, is of great importance in those types of studies [30, 31]. Therefore, patients with various mental and substance abuse diseases—to begin with—do not often volunteer and secondly, are generally excluded from clinical trials. This may improve the abstinence results of randomized trials and underestimate the significance of addictions in COPD. In clinical practice these patients may benefit from tight collaboration between their pulmonologist and psychiatrist when their cessation program is tailored. Comanagement of depression and smoking cessation has already shown to be feasible and safe. It increased the quit attempts and the success in cessation did not increase the risk of exacerbation of depression [32].

In addition to above-mentioned strengths our observational study design included also weaknesses. The cumulative clinical data was mainly based on retrospective medical records which were not originally produced for research purposes and therefore prevented us from the further dissection of the severity of psychiatric conditions or alcohol abuse. The smoking history reported by the patients in different cross sectional time points showed some inconsistencies. It seemed difficult for patients to remember when they had started and stopped smoking especially when that had happened a long time ago. At COPD diagnosis and during active treatment the data was more current and thus more adequate. That is why the data should be considered more like rough estimates rather than precise knowledge of patients pack years, starting and quitting dates. The Finnish Current Care Guidelines on Tobacco dependence and cessation and on COPD treatment are followed in the Finnish hospitals, but unfortunately no validated stopping program has been used [33]. In the Turku University Central Hospital patients were systematically referred to an individual or group counseling given by a registered nurse that improved the quit rates (66% versus 54%) suggesting that greater counseling efforts pay off among the COPD patients.

The present study emphasizes the heterogeneous clinical background of COPD patients, the role of coexisting diseases on smoking cessation, and the importance of quitting in the prognosis of COPD. New treatment options that comprehensively recognize patients' mental health and addiction profiles, and evaluate the patient's need of psychiatric help and/or medication, may benefit certain patient groups in their smoking cessation and could reduce mortality in these patient groups.

## Figures and Tables

**Table 1:** Demographics and clinical characteristics of quitters and nonquitters.

Characteristics	All	Quitters^1^	Nonquitters	*P* value
	*N* = 739 (%)	*N* = 433 (%)	*N* = 306 (%)	
Men	473 (64)	285 (60.3)	188 (39.7)	NS
Women	266 (36)	148 (55.6)	118 (44.4)	
Age, mean (±SD) in years	64.0 (6.8)	65.3 (6.6)	62.3 (6.8)	**<0.001**
Duration of COPD mean (±SD) in years	5.5 (4.1)	5.8 (4.4)	5.2 (3.8)	NS
BMI (kg/m^2^) (±SD)	26.5 (5.3)	26.5 (5.2)	26.5 (5.5)	NS
Pack years (±SD)	41 (23)	43 (24)	38 (22)	**0.01**
Baseline mean (±SD)				
FEV_1_ % predicted	58.4 (18.9)	56.3 (18.4)	61.3 (19.3)	**<0.001**
FVC % predicted	73.6 (18.1)	72.6 (17.9)	74.9 (18.3)	**0.04**
FEV_1_/FVC %	64.0 (13.9)	62.7 (14.4)	65.8 (13.0)	**0.002**
After bronchodilatation mean (±SD)				
FEV_1_ % predicted	62.1 (18.3)	60.3 (18.1)	64.4 (18.4)	**0.004**
FVC % predicted	77.8 (17.3)	77.4 (17.2)	78.4 (17.3)	NS
FEV_1_/FVC %	64.4 (13.9)	63.0 (14.4)	66.4 (13.0)	**0.002**
*N* (%) of patients with				
FEV_1_ % >80	87 (12.0)	41 (9.6)	46 (15.4)	**0.005 for trend**
FEV_1_ % 65–80	191 (26.4)	100 (23.5)	91 (30.5)	
FEV_1_ % 40–64	309 (42.7)	196 (46.1)	113 (37.9)	
FEV_1_% <40	139 (18.8)	88 (20.7)	48 (16.1)	
General-health-related QoL score (range 0-1) (±SD)	0.79 (0.11)	0.80 (0.10)	0.78 (0.12)	**0.05**
Airway specific QoL score (range 20–0) (±SD)	8.25 (5.03)	7.98 (4.77)	8.63 (5.35)	NS
Comorbidities *N* (%)				
Cardiovascular diseases	216 (29.2)	141 (32.6)	75 (24.5)	**0.02**
Diabetes	121 (16.4)	72 (16.7)	49 (16.1)	NS
Alcohol abuse	129 (17.8)	49 (11.5)	80 (26.6)	**<0.001**
Psychiatric disorder	177 (24.1)	75 (17.5)	102 (33.6)	**<0.001**
Cancer	78 (10.6)	49 (11.3)	29 (9.5)	NS
Deceased during the followup	114 (15.3)	52 (12.2)	62 (20.7)	**0.002**

^1^Early and late quitters combined.

**Table 2:** Independent risk factors for failure in smoking cessation by multivariate logistic regression analysis.

Variables	Adjusted OR	95% CI
Female gender	1.08	0.75–1.55
Aging by one year	**0.96**	**0.93**–0.99**
FEV_1_ % of predicted		
>80	1.00	
65–80	0.84	0.48–1.46
40–64	0.60	0.35–1.02
<40	0.59	0.32–1.09
Number of pack years increased by one	0.99	0.99–1.00
Alcohol abuse	**2.12**	**1.35**–3.34**
Psychiatric disorder	**1.83**	1.23–2.71**
Cardiovascular diseases	0.78	0.53–1.15
Diabetes	1.07	0.68–1.68
Cancer	0.85	0.49–1.47

Response (nonquitter = 1, quitter = 0) ^∗∗^ *P* ≤ 0.01 ^∗^ *P* ≤ 0.05.

**Table 3:** Underlining clinical characteristics that predict mortality of the COPD patients during the four-year followup.

Variables	Patient* N* = 739	Deceased %	Crude OR	95% CI	*P* value	Adjusted OR	95% CI	*P* value
Aging by one year			**1.05**	**1.02–1.09**	**0.002**	**1.07**	**1.03–1.11**	**0.001**
Gender								
Male	466	18.0	1.00			1.00		
Female	259	11.6	**0.60**	**0.38–0.93**	**0.022**	0.81	0.48–1.435	NS
Smoking cessation								
Yes	426	12.2	1.00			1.00		
No	299	20.7	**1.88**	**1.26–2.82**	**0.002**	**2.50**	1.55–4.03	**<0.001**
Cardiovascular diseases								
No	514	13.0	1.00			1.00		
Yes	211	22.3	**1.91**	**1.26–2.89**	**0.002**	1.52	0.94–2.46	NS
Diabetes								
No	606	14.5	1.00			1.00		
Yes	117	22.8	**1.68**	**1.03–2.75**	**0.036**	1.38	0.79–2.43	NS
Alcohol abuse								
No	584	13.7	1.00			1.00		
Yes	128	24.2	**2.01**	**1.26–3.22**	**0.003**	**2.03**	**1.14–3.61**	**0.016**
Psychiatric condition								
No	544	14.9	1.00			1.00		
Yes	175	17.1	1.18	0.75–1.87	NS	1.32	0.77–2.26	NS
Cancer								
No	649	13.7	1.00			1.00		
Yes	74	32.4	**3.02**	**1.77–5.16**	**<0.001**	**3.08**	**1.70–5.59**	**<0.001**
FEV_1_ % of predicted								
>80%	87	5.7	1.00			1.00		
80–65%	191	9.9	1.81	0.65–5.02	NS	2.12	0.68–6.67	NS
65–40%	306	16.5	**3.24**	**1.25–8.40**	**0.015**	**3.76**	**1.27–11.15**	**0.017**
<40%	136	28.7	**6.59**	**2.48–17.51**	**<0.001**	**8.77**	**2.88–26.68**	**<0.001**

Response (dead = 1, alive = 0).
